# Speech and language therapists' insights into severity of speech sound disorders in children for developing the speech sound disorder severity construct

**DOI:** 10.1111/1460-6984.70022

**Published:** 2025-03-26

**Authors:** Anniek van Doornik, Marlies Welbie, Sharynne McLeod, Ellen Gerrits, Hayo Terband

**Affiliations:** ^1^ Research group Speech and Language Therapy – Participation through Communication HU University of Applied Sciences, Heidelberglaan 7 Utrecht The Netherlands; ^2^ UIL‐OTS Utrecht University, Trans 10 Utrecht Utrecht The Netherlands; ^3^ Research Group of Research Competence HU University of Applied Sciences, Padualaan 97 Utrecht The Netherlands; ^4^ Early Childhood Interdisciplinary Research Group | Education Charles Sturt University, Panorama Avenue Bathurst NSW Australia; ^5^ Department of Communication Sciences and Disorders | Speech Sensorimotor Development Lab University of Iowa, 250 Hawkins Dr. Iowa City Iowa USA

**Keywords:** child's perception, intelligibility, qualitative, severity, speech assessment, speech sound disorders, SSDSC

## Abstract

**Background:**

Children with speech sound disorders (SSD) are at higher risk of communication breakdown, but the impact of having an SSD may vary from child to child. Determining the severity of SSD helps speech‐language therapists (SLTs) to recognise the extent of the problem and to identify and prioritise children who require intervention.

**Aims:**

This study aimed to identify severity factors for SSD in order to develop a multifactorial Speech Sound Disorder Severity Construct (SSDSC) using SLTs’ views and the International Classification of Functioning, Disability and Health (ICF).

**Method:**

In an explorative five‐staged qualitative study, the research question was answered: ‘How do SLTs determine the severity of SSD in children?’. A total of 91 SLTs from The Netherlands participated in data collection and analysis. The iterative process included three different qualitative research methodologies (thematic analysis [TA], constructivist grounded theory [CGT] and content analysis [CA]) to ensure validation of the results by means of method triangulation.

**Results:**

SLTs considered nine themes: intelligibility, speech accuracy, persistence, the child's perception, impact, communicative participation, concomitant factors, professional point of view, and environmental factors. The themes were summarised in three main severity factors: (I) Speech accuracy, (II) The child's perception of the impact of their speech, and (III) Intelligibility in communication. Other severity factors were concomitant factors and impact. Expertise and support were identified as facilitators or barriers that may worsen or relieve the severity of SSD.

**Conclusions:**

This study highlights the need for SLTs to rethink how they think about severity as a simplistic construct reflecting only speech accuracy. It is recommended that a broader holistic approach to measuring severity is adopted.

**WHAT THIS PAPER ADDS:**

**What is already known on the subject**

- Although there are several proposals aiming to define the severity of SSD, the extent to which these align with clinical practice is not well understood. In recent years, speech accuracy and other factors such as intelligibility have been suggested as possible indicators of SSD severity. Flipsen et al. (2005) concluded that experienced clinicians evaluated the number, type and consistency of speech errors as well as intelligibility, considering articulatory competence at both the segmental and whole word level when determining severity. Enderby et al. (2013) developed ICF‐based Therapy Outcome Measures (TOMs), including a TOM for phonological disorder. Although there is evidence in the literature that multiple factors should be considered when determining severity, there is still no clear guidance on this. In this discussion, the perspective of SLTs is an important contribution to the development of a measure of severity, but is lacking in the known literature.

**What this paper adds to the existing knowledge**

- As a first step, this study examines SLT's perspectives on how they define and measure severity of SSD, and determines how those views align with the ICF in order to develop a severity construct that can be further tested and validated. Using the views of SLTs and the ICF, this qualitative study resulted in the multifactorial SSDSC.

**What are the potential or actual clinical implications of this work?**

- The practical experience of SLTs, combined with what is known from the literature, provides insight into the different factors that may contribute to severity of SSD. These factors may be considered in developing a measure of SSD severity in the future.

## INTRODUCTION

Successful speech, language and communication in daily life is important for full participation in society. Children with speech sound disorders (SSD) are at higher risk of communication breakdown because their speech is less accurate and less intelligible compared with the speech of children with typical development (TD). The impact of having an SSD may vary from child to child and may affect their participation at school, at home, or in social life. To support children with SSD, it is important to gain a nuanced view of the nature and the impact of SSD. Traditional assessments by speech‐language therapists (SLTs) of children's speech focused on production in single words and connected speech (e.g., McLeod & Baker, 2014; Wikse Barrow et al., 2021). However, SLTs have been encouraged to undertake holistic assessments (e.g., Cronin et al., 2020) that encompass all aspects of the International Classification of Functioning, Disability, and Health (ICF, World Health Organization, 2001). Accordingly, the World Health Organization (2001) recommends assessment of the severity as well as the nature of disabilities. Consequently, SLTs are encouraged to diagnose the severity of SSD in children, which is included in SLTs’ clinical guidelines (e.g., Nederlandse Vereniging voor Logopedie en Foniatrie, 2019). Determining severity of SSD helps SLTs to recognise the extent of the problem and to identify and prioritise children who require intervention. Understanding severity, therefore, is important for diagnosis, prognosis, amount of intervention, and prioritisation of services. Severity as an outcome measure in therapy is important to quantify its effect, monitor progression and learn about the effectiveness of intervention.

### Severity measures

Although there are several ways to define the severity of SSD, the extent to which these align with clinical practices is not well understood. Speech accuracy and intelligibility have been used as severity measures for SSD (Donicht et al., 2009; Flipsen et al., 2005; Garrett & Moran, 1992; Gordon‐Brannan & Hodson, 2000; Lousada et al., 2014; Newbold et al., 2013; Shriberg & Kwiatkowski, 1982).

Speech accuracy has been described using a variety of outcome measures, such as percentage of consonants correct (PCC), percentage of vowels correct (PVC), and percentage of phonemes correct (PPC) (Shriberg, 1993; Shriberg et al., 1997). However, many studies have designated PCC as a robust outcome measure to describe speech accuracy (Shriberg & Kwiatkowski, 1982; Garrett & Moran, 1992; Newbold et al., 2013; McLeod & Baker, 2014). Barrozo et al. (2017), and Shriberg et al. (1997) demonstrated that PCC was effective in discriminating and identifying children with and without SSD. Shriberg and Kwiatkowski (1982) described four classification boundaries based on PCC outlining severity of speech accuracy from *mild* to *severe*. Johnson et al. (2004) also used PCC as a severity measure and recommended using imitation for a time‐efficient calculation of PCC to determine severity of SSD. Other researchers described the limitations of using PCC as a severity measure (Dodd, 2013). Using PCC to define boundaries of *mild*, *moderate* or *severe* has been recognised as problematic, because boundaries were difficult to validate (Flipsen et al., 2005). Therefore, when PCC is used as a severity measure, it should be related to the age of the child because of the developmental nature of speech accuracy (McLeod & Crowe, 2018). Other measures have been suggested such as phonological mean length of utterance (PMLU; Beers et al., 2019; Ingram, 2002), feature complexity (Brancalioni et al., 2012), or the number of phonological process errors (Torres et al., 2020).

Intelligibility has also been recommended to describe the nature and severity of SSD, and for the evaluation of therapy (Donicht et al., 2009; Gordon‐Brannan & Hodson, 2000; Lousada et al., 2014). Kent et al. (1994) described 19 ways to test intelligibility, such as phonetic contrast analysis, phonological process analysis, word identification tests, phonetic indices derived from continuous speech scoring, scaling of continuous speech, and traditional word‐level analysis of continuous speech. Miller (2013) also stressed the importance of intelligibility testing and recommended combining signal‐dependent and signal‐independent intelligibility measures for clinical diagnosis and outcome measurement. Intelligibility as a signal‐dependent concept is based solely on the sound signals of the spoken message; whereas, intelligibility as signal‐independent intelligibility measure includes the verbal and non‐verbal context of the message. McLeod (2020) found a correlation across languages evaluating the validity and reliability of the Intelligibility in Context Scale (ICS). A recent study by Chenausky et al. (2022) evaluating severity and intelligibility of children with childhood apraxia of speech (CAS), showed that there was a high correlation between the estimations of intelligibility and perceived single‐word speech severity. Despite attempts to find a single, unified measure of severity, several studies point to a holistic approach to determining the severity of SSD. For example, Flipsen et al. (2005) evaluated a variety of segmental and whole word measures, including PCC and intelligibility. They concluded that experienced clinicians evaluated the number, type and consistency of speech errors as well as intelligibility, considering articulatory competence at both the segmental and whole word level when determining severity. McCormack and McLeod et al. (2010) recommended a holistic approach during the assessment phase, including an assessment of the child's and parent's listening problems and frustration, in addition to the assessment of speech accuracy.

### Comorbidity

Comorbid or underlying factors also may determine the severity of SSD. SSD may occur together with developmental language disorder (DLD), literacy difficulties, oral motor difficulties, hearing loss, voice difficulties, and stuttering. Although the concept of severity was not defined, Dodd (2014) concluded that associated abilities such as impaired perceptual and motor control or phonological working memory, phonological awareness or executive function may impact SSD, although the direction of causality is unclear. Other studies show that severity of SSD increased in children with comorbid DLD (Torres et al., 2020) or auditory processing disorder (Vilela et al., 2016). Chilosi et al. (2022) found that children with CAS often have comorbid expressive and receptive DLD, possibly because motor speech problems limit the production of longer speech chains and therefore may affect morphology. On the other hand, it has been suggested that improved language skills may improve speech sequencing skills. In another study, CAS with other comorbid diagnoses (e.g., galactosemia) resulted in lower communication profiles and smaller phonemic repertoire. Thus, comorbidity again had a negative impact on SSD (Iuzzini‐Seigel et al., 2022).

### Severity in light of the ICF

SLTs worldwide use the International Classification of Functioning, Disability and Health (ICF, World Health Organization, 2001) as a framework to describe the functioning of their clients (e.g., American Speech‐Language Hearing Association). The ICF offers a holistic, conceptual framework and comprises five domains: Body Functions, Body Structures, Activities and Participation, Personal Factors, and Environmental Factors. The Personal and Environmental factors influence how disability is experienced by the individual and contribute to the understanding of functioning and disability as described by Body Functions, Body Structures, Activities and Participation.

The World Health Organization (2001) uses four qualifiers for defining severity: *mild, moderate*, *severe* and *complete* and explicitly stresses the importance of qualifiers on every domain of the ICF conceptual framework when indicating the severity of a health condition. Words such as ‘no’, ‘complete’, and ‘moderate’ are used in combination with a determiner to qualify the problems. In the domain Body Functions, ‘impairment’ is used to describe the severity; ‘performance’ is used to qualify Activities and Participation, and ‘barriers and facilitators’ are used to qualify the Environmental and Personal Factors (World Health Organization, 2017). Threats (2008) discussed the application of severity measures to different domains of the ICF. He argued that different elements within the ICF require different assessment tools and different interpretations of severity. His conclusion is that it is important to measure how quality of life is affected by the communication disorder and that health professionals, such as SLTs, are responsible for developing appropriate severity measures. McCormack, McLeod, Harrison, et al. (2010) evaluated the use of the domains of Activities and Participation of the ICF—Children and Youth (ICF‐CY, World Health Organization, 2001) in relation to children with SSD. They concluded that Activities and Participation should be considered in understanding the impact of SSD on children. These ideas were supported by Feeney et al. (2012), who stated that health‐related quality of life is a multidimensional concept that encompasses numerous domains of functioning.

John and Enderby (2000) used the ICF as the basis for the development of a holistic outcome measure for interventions, the Therapy Outcome Measure (TOM). They developed TOMS for different conditions, including phonological disorders. These TOMS are based on a severity rating that includes impairment, activity, participation and wellbeing/distress. For phonological disorders, they linked speech accuracy to impairment, intelligibility to activity, fulfilling a social role in general to participation, and levels of anxiety, depression, frustration, etc. to wellbeing/distress. These characteristics would be involved in change through therapy, based on data from the Read coding system used within the UK National Health Service (John, 2011). The construct validity of this TOM was examined by Roulstone et al. (2004) and showed that the phonological error score and the TOM impairment score were related, but nothing was said about the other characteristics. However, as an outcome measure of therapy, a TOM is not suitable for a single assessment. There is always a need for comparison with an initial assessment and with the therapy being provided.

Although the use of TOMS is consistent with the view that severity requires a holistic description, it is not known how the selection of factors fits with SLTs’ views on factors that influence severity. In addition, the use of a TOM is based on professional judgement, whereas the inclusion of multiple views, such as the judgement of parents and children themselves, is important, too.

There are benefits to involving health professionals in research. Hanney et al. (2013) concluded from their three‐stage review that when clinicians and health care organisations are involved in research, there is a greater chance of a positive impact on health care performance. In their systematic review, Noar et al. (2023) showed that Communities of practice (CoPs), such as a group of health professionals, contributed to better research outcomes for clinical practice. SLTs are considered to be experts in defining severity because of their specific knowledge about speech development and their expertise in diagnosing and treating SSD in children. It is therefore evident to work with SLTs in this study. Regarding the children's perspectives, in a recent study, Zerbeto et al. (2023) found that children with speech and language disorders had different perceptions of their own speech and difficulties in the ICF domains of Body Functions, Activities and Participation, and Environmental Factors 6 months after SLT intervention. Thus, SSD affects aspects of functioning at different domains, providing a rationale for a multifactorial severity construct.

As SLTs worldwide use the multifactorial ICF framework in their daily practice, the current study used the ICF framework to inform the construct of severity for children with SSD (cf. McLeod & McCormack, 2007). That is, Body Functions include speech functions, including articulation of vowels and consonants, timing and intonation of speech, but also mental functions such as temperament, and coping strategies. Activities include speaking and conversation, and Participation refers to communication in daily life. Personal and Environmental Factors as barriers or facilitators may also impact the severity of SSD, and are seen as influencing severity factors, for example parental support. Identifying factors that contribute to the severity of SSD can improve service delivery and outcomes for children with SSD.

### Aim of the study

It is clear from the literature that a specific severity measure focused on speech accuracy does not capture the multiple factors that may influence the impact of SSD on a child; specific measures are not holistic. Secondly, regardless of whether a severity measure is specific or holistic, if it is based on clinical judgements of severity without a universal or agreed understanding of the meaning of the construct by SLTs, severity measures may be prone to bias or compromised inter‐judge reliability. Therefore, the purpose of this study was to examine SLTs’ perspectives on how they define or measure SSD severity in their daily practice, and secondly, to determine how those views align with the ICF, resulting in a multifactorial Speech Sound Disorder Severity Construct (SSDSC). An exploratory five stage qualitative study was undertaken to address the research question: ‘How do SLTs determine the severity of SSD in children?’. Data were collected and analysed in an iterative process following three different qualitative research methodologies (thematic analysis [TA], constructivist grounded theory [CGT] and content analysis [CA]) to ensure validation of the results by means of method triangulation. Every stage was led by a specific research question which helped to answer the main research question:

- Stage I: What severity indicators are mentioned by SLTs in relation to SSD?
- Stage II: How is the severity of SSD determined by SLTs in daily practice?
- Stage III: How can the outcomes from Stage I and II be merged to one set of themes?
- Stage IV: How do the items on the severity indicators list compare to speech‐related ICF codes?
- Stage V: How can the SLTs’ views and the ICF codes be integrated in a SSDSC?

The Standards for Reporting Qualitative Research checklist (SRQR; O'Brien et al., 2014) was used as a guideline for reporting the study. In this study, an inclusive definition of SSD was used and SLTs were asked to think about all types of SSD (i.e., articulation, phonology, CAS, dysarthria). Therefore, the SSDSC will apply to all children with SSD.

## METHOD

### Overview of the study

A qualitative research approach was undertaken with an iterative research process that comprised five stages in which data were alternately or simultaneously collected and analysed (Table 1; Appendix I‐V). The paradigm underlying this study is that reality consists of multiple realities. The purpose of this research is to explore and interpret these realities in order to develop a theory of the severity of SSD. In this case, the theory will be translated into a multifactorial model (SSDSC) that can be used to determine the severity of an SSD in the future. The main research methodology used in this study is based on Grounded Theory, which led to the proposed SSDSC. Methods have been followed that are in line with CGT (Bryant & Charmaz, 2019; Letts et al., 2007; Lyons & McAllister, 2019; Metelski et al., 2021), where the starting point is that the researcher interprets the meaning of the experiences together with the participants and constructs the model together, and is based on mutual input and mutual interpretation of data. In this case, a reality about determining the severity of SSD was constructed together with the SLTs during conversations in which the SLTs shared their practical experiences. We assume that SLTs have explicit and implicit knowledge about determining severity, that they can make this implicit knowledge explicit in group discussions in which insights are co‐created. The beliefs, values and perspectives of the SLTs and the first author AD, who is also an SLT, were explored in Stage II, by collecting and analysing data during a focus group.

**TABLE 1:** Overview of the research process.

Step	Activity	Description	Objective	Result	Rigour
Stage I TA: First inventory of severity indicators of SSD					
TA 1	Severity indicators inventory	Familiarisation	Data collection and exploration	Severity indicators list	–
TA 2	–	Initial coding	Data analysis	Codes	Investigator triangulation
TA 3	Classification assignment	Searching for themes by researcher and participants	Data analysis	Potential themes	Investigator triangulation
	Counting	–	–	–	Method triangulation
TA 4	Consensus workshop	Reviewing themes by consensus on potential themes	Data analysis	Reviewed themes	Investigator triangulation
Stage II CGT: Exploration of SLTs’ daily practice towards to the determination of severity of SSD					
CGT 1	–	Familiarisation	preparation	Topic list for focus group discussion	–
CGT 2a	Focus group	Focus group discussion	Data collection	Quotes	Method triangulation
					Prolonged engagement
CGT 2b	–	Reflective discussion	Data collection	Quotes	Data triangulation
					Member check
CGT 2c	–	Initial open coding	Data analysis	Codes	Method triangulation
					Coding Loop
					Investigator triangulation
CGT 3	–	Focused coding	Data analysis	Focused codes	–
Stage III TA: Merging the results of Stage I and Stage II					
TA 5	–	Defining themes	Data analysis	Themes	Saturation
					Method triangulation
Stage IV CA: Validation					
CA 1	–	Preparation phase: familiarisation	Data analysis	Categorization matrix from ICF code system	Dependability
CA 2	–	Organising by a deductive analysis on the severity indicators using ICF codes	Data analysis	ICF severity codes	Investigator triangulation by data coder check
Stage V CGT: Integration					
CGT 4	–	Theoretical coding for Theory construction	Data analysis	Themes	Transferability
					Confirmability
CGT 5	–	Synthesis of the results	Data analysis	Co‐constructed multifactorial SSDSC. Identification of severity factors, facilitators & barriers.	–

Abbreviations: CA, content analysis; CGT, constructivist grounded theory; ICF, International Classification of Functioning, Disability and Health; SLTs, speech‐language therapists; SSDSC, Speech Sound Disorder Severity Construct; TA, thematic analysis.

The CGT is supported by two methods from a different approach to qualitative research. First of all, according to Braun and Clark (2018), a method suitable for small q TA was applied in Stage I. This method, which stems from a positivist philosophy, ensures a structured approach to coding, with an emphasis on ensuring reliability and accuracy of coding. TA methods were used in the severity indicators inventory, a classification assignment, and a consensus workshop. The TA was concluded in Stage III by combining the preliminary results from Stage I (TA) and Stage II (CGT). The TA provides more scope for analysing a list of severity indicators mentioned by SLTs in relation to the severity of SSD. This made it possible to find themes in the list in a structured way. Finally, these findings were incorporated into the further CGT analysis.

In addition to the TA, CA methods (Elo & Kyngäs, 2008; Hsieh & Shannon, 2005) were used in the deductive analysis in Stage IV when comparing the items in the severity indicators list with a selection of ICF codes (World Health Organization, 2017). The reason for carrying out a CA is that SLTs use the ICF in their daily practice. This is a binding framework that is difficult to incorporate into the CGT's working method, as it guides and predetermines the codes with which the SLTs' responses are to be compared. The central question in the CA was how the identified severity indicators relate to the ICF codes. This is important for the applicability of the model in practice. The results of the TA and CA were finally included as data points in the CGT analysis in step V, in which the results were reconsidered, combined and interpreted.

The combination of qualitative methods resulted in methodological triangulation of multiple data sources and allowed the researchers to find saturation in the data. The study was conducted in an organic and intuitive manner. To ensure investigator triangulation, interpretation of the data was constantly discussed with SLTs and the authors who had expertise in SSD, motor speech disorders, qualitative research, and SLT research.

The activities in this study were led by the first author AD in the role of moderator. She is a speech and language therapist and lecturer at a SLT bachelor programme. She was assisted by a final (fourth) year SLT bachelor student. The assistant took notes on the process and interaction and transcribed the video recordings verbatim. The second author is a physiotherapist, senior researcher and experienced in qualitative research. She has supervised the qualitative analysis. The third and fourth authors are also SLTs and senior researchers in speech and language disorders in children. The last author is a phonetician and senior in fundamental speech analysis. The results were discussed with all authors.

In total 91 SLTs from the Netherlands participated in Stage I and II in data collection and data analysis. Participants were selected using convenience and expert opinion sampling. All questions and answers were in Dutch and translated into English for this paper.

### Stage I: First inventory of SSD severity indicators

The study began with SLTs creating a broad inventory of SSD severity indicators SLT. The research question in Stage I was: What severity indicators are mentioned by SLTs in relation to SSD? An overview of Stage I is provided in Appendix I.

#### Stage I participants

A total of 91 SLTs were participants in this study: 68 participants contributed to the data collection, and 23 contributed to data analysis. All participants spoke Dutch and worked as an SLT in The Netherlands. First, 55 participants were invited (50 SLTs and 5 SLT bachelor students) using convenience sampling and including attendees at the yearly conference of the Dutch SLT Association. Most attendees at the conference worked in SLT practices and had experience in working with children with SSD. An additional 14 SLTs were recruited because they worked in special toddler groups and special schools for children with SSD or/and DLD. SLTs in these specialised organisations are highly experienced in delivering services to children with severe SSD and DLD, and see only children with SSD and/or DLD in their daily practice. SLTs in SLT practices have a mixed caseload, with children with SSD and/or DLD making up the largest group (Priester et al., 2009). The combination of participants from SLT practices and SLTs from specialised institutions ensured that experience with the whole spectrum of mild to severe disorders was represented in this study.

In Stage I, 23 participants also contributed to the data analysis. Two groups of participants were involved in two activities: a classification assignment and a consensus workshop. First, 10 SLTs formed a focus group and participated in the classification assignment. The SLTs were asked to participate via a Facebook group for Dutch SLTs. Participants were selected by purposive sampling, aiming at maximum variation in age, years of experience (from 1.5 to 39 years), SLT educational grade (BSc or MSc), and work setting (SLT practice, primary schools, special education, and expertise centres). Table 2 provides demographic information about the focus group participants.

**TABLE 2:** Participants’ characteristics in the focus group and classification assignment (*n* = 10).

Pseudonym	Age	Qualifications^a^	Work setting^b^	Years of experience
Merel	23	SLT, MSc SLP	SLT's practice	1.5
Lisa	24	SLT, MSc. CCAP	SLT's practice	1.5
Denise	28	SLT	SLT's practice	1.5
Iris	28	SLT, MSc SLP	SLT's practice	6
Esther	30	SLT, MSc SLP	EC	6
Anna	30	SLT, GL	HSC	9
Jolanda	40	SLT	EC, SS, PE	19
Sandra	45	SLT	SLT's practice	23
Carolien	52	SLT	SLT's practice	29
Karin	60	SLT	SLT's practice	39

^a^ SLT, Speech and Language Therapist (In the Netherlands, Bachelor SLTs are certified practitioners); MSc SLP, Master of Speech and Language Pathology; GL, General Linguistics; MSc. CCAP, Master of Clinical Child and Adolescent Psychology.

^b^ EC, Expertise Centre for children with DLD and SSD; SS, Special School for children with DLD and SSD; PE, primary education; HCS, Hearing and Speech Centre.

Second, for the consensus workshop, 13 new participants were recruited at the yearly symposium of the university's research group. The participants were aware of the aim of the research as described in the symposium invitation and were asked to provide informed consent at the registration desk (Table 3).

**TABLE 3:** Participants’ characteristics in the consensus workshop (*n* = 13).

Participant in the consensus workshop	Age	Qualifications^a^	Work setting^b^	Years of experience
1	54	SLT	SLT's practice	30
2	61	SLT	SLT's practice + SS	36
3	49	SLT, MSc SLP	HCS	26
4	26	SLT	SLT's practice	2
5	54	SLT, TP	SS	30
6	21	Undergraduate BSc	Student	0
7	22	Undergraduate BSc	Student	0
8	24	SLT	SS	1
9	56	SLT, TP	SLT's practice	24
10	26	SLT, MSEN	SLT's practice + SS	6
11	52	SLT	HCS	28
12	46	SLT	EC	23
13	43	SLT, PROMPT trained	HCS	21

^a^ SLT, Speech and Language Therapist (In the Netherlands, Bachelor SLTs are certified practitioners); MSc SLP, Master of Speech and Language Pathology; TP, teacher in primary schools; MSEN, Master of Special Educational Needs.

^b^ EC, Expertise Centre for children with DLD and SSD; SS, Special School for children with DLD and SSD; PE, primary education; HCS, Hearing and Speech Centre.

#### Stage I procedure

Participants of the national SLT conference and two network meetings were informed about the aim of the study and provided consent to be involved in the research. They were asked to write their answer to the following question on coloured post‐it notes: ‘What determines the severity of a speech sound disorder in children aged 4–6 years?’. Each participant was given one red post‐it note to write down their first response and placed their response on the jacket of a life‐sized image of a child. Other responses were written on blue and white post‐its and also placed on the jacket. Anonymity of the participants was protected because names were not recorded.

#### Stage I data analysis

The Stage I data were analysed in five steps using TA methods (Braun & Clarke, 2021). Themes which represent patterns in the data, were identified by the first author and the participants. TA terminology was used to describe the data in five steps:

- **Data familiarisation**: Handwritten data were transcribed in Excel (Microsoft, 2019) into a severity indicators list, and identical wordings were merged.
- **Generating codes**: Items in the severity indicators list were systematically coded by the first author and checked by members of the research group.
- **Searching for themes**: The codes were first collated into potential themes by the first author. The search for themes was then continued in co‐creation with participants during the classification assignment. The participants worked in pairs to categorise the items from the severity indicators list, relying on their own frames of reference. The moderator (first author) and research assistant asked for the background ideas of the categories and took notes. Next, the categories created by the first author and the participants were compared and potential themes were created. Allocation of a severity indicator to a potential theme depended on counting: if 4/5 SLT‐pairs had categorised in the same way, the severity indicator was assigned to the potential theme.
- **Reviewing themes**: The severity indicators that had not been allocated to a potential theme were reviewed by 14 participants in a consensus workshop. 100% consensus was required to assign the severity indicator to a potential theme. The consensus workshop yielded a list of seven reviewed themes.
- **Defining themes**: Defining themes is described in Stage III, since it combines the results from Stage I and II.

### Stage II: Further exploration of the concept of severity

The aim of Stage II was to explore: ‘How is the severity of a speech sound disorder determined by SLTs in daily practice?’ The philosophical assumption was that SLTs have explicit and tacit knowledge about the severity of SSD in children. Therefore, both explicit and tacit knowledge was elicited in different activities during a focus group session.

Data were collected during the focus group session, first during a group interview, then during a reflective discussion. These different methods verified whether the data represented credible views, and whether the interpretation was valid. During the focus group interview, the SLTs were not aware of the outcome of the severity inventory and spoke about their own experiences in daily practice without prior information. Next, during the reflective discussion, they were aware of the results in Stage I and could match their views to the items of the severity indicators list. This procedure helped identify characteristics and elements that were most relevant to the determination of severity in children with SSD. An overview of Stage II is provided in Appendix II.

#### Stage II participants

The same SLTs who contributed to the classification assignment in Stage I (*n* = 10) participated in the two focus group discussions. As described in Stage I, participants were selected by purposive sampling, aiming at maximum variation in age, years of experience, and educational grade (Table 2).

#### Stage II procedure

The focus group interview and the reflective discussion were prepared using the Centraal BegeleidingsOrgaan (CBO) guidelines for focus groups (CBO, 2004). The first activity of the focus group session was a group interview. The interview was structured using the severity indicators list, literature from the introduction of this paper, and the clinical experience of the moderator. The focus group session was completed with a reflective discussion. The reflective discussion aimed to recognise, confirm, and clarify severity indicators from the group interview and from the severity indicators list. Theoretical saturation was achieved when the focus group participants reported that they could not think of new information.

#### Stage II data analysis

In Stage II, CGT was applied and CGT terminology was used to describe the analysis. The CTG procedure comprised four steps:

- **Familiarisation**: As a first step of familiarisation, the items on the severity indicators list from Stage I were added to the topic list for the interview guide. The group interview yielded discussion quotes, and the reflective discussion yielded reflective quotes.
- **Open coding**: The quotes were analysed in step 2 open coding, in an inductive analysis in Atlas.ti (8.4.19.0) (Atlas.ti Scientific Software Development GmBH, 2019). A data‐coder check with all authors validated the open codes, resulting in a list of codes, (Appendix).
- **Focused coding**: Focused coding, aimed at restructuring the data and identifying underlying concepts in the dataset. The analysis resulted in a set of focused codes that represented the indicators used in the SLTs’ daily practice to determine the severity of SSD.
- **Theoretical coding**: The step of theoretical coding is described in Stage V.

### Stage III: Merging results Stage I and II

Stage III comprised the final step (5) of the TA and was led by the question: ‘How can the outcomes from Stage I and II be merged to one set of themes?’. An overview of Stage III is provided in Appendix III.

#### Stage III analysis

The results from the severity inventory (Stage I reviewed themes) and the results from the focus group (Stage II focused codes) were merged in order to define themes. The reviewed themes and the focused codes were compared with the severity indicators and quotes. Consequently, themes were defined using words that covered the content of the reviewed themes and focused codes.

### Stage IV: Validation

In Stage IV the views on severity of SSD by SLTs were validated by answering the research question ‘How do the items on the severity indicators list compare to speech‐related ICF codes?’ The ICF‐system comprises a framework and ICF codes (e.g., B320, Articulation functions). The codes are used to classify functional abilities, where every code is linked to a domain of the ICF framework. The ICF codes provide a standard language for the definition of health and disability. By comparing the answers from the SLTs to the ICF codes, it validated the fit with the ICF framework. Both the third and first author were experienced in using the ICF codes, and applying the framework to SLT practice. The first author contributed to the implementation of the ICF for SLTs in The Netherlands, and the third author in Australia.

#### Stage IV analysis

A deductive analysis using methods from CA (Elo & Kyngäs, 2008; Hsieh & Shannon, 2005) was carried out and CA terminology was used to describe the analysis in two phases:

- **Preparation**: During the preparation phase, the severity indicators list was selected as the unit of analysis. The comparisons in Stage III revealed that the items on the severity indicators list were highly comparable to the quotes from the focus groups. It was hypothesised that by comparing the items from the severity indicators list and speech‐related ICF codes that a representative set of ICF severity codes would emerge.
- **Organising**: In order to create a categorisation matrix, speech‐related ICF codes were selected by the first author from the ICF browser (World Health Organization, 2017), drawing on relevant literature (e.g., McLeod & Baker, 2014; McLeod & McCormack, 2007; McLeod & Threats, 2008). The speech‐related ICF codes were uploaded in Atlas.ti (8.4.19.0) (Atlas.ti Scientific Software Development GmBH, 2019). Consequently, as a final step in the organising phase, the speech‐related ICF codes were matched with the items from the severity indicators list by the first author. The deductive analysis resulted in a selection labelled the ICF severity codes that matched the items on the severity indicators list. The accuracy of the application of the speech‐related ICF codes to the data was anchored by investigator triangulation during research group meetings where the interpretation of the codes was discussed. An overview of Stage IV is provided in Appendix III.

### Stage V: Integration

In Stage V all results from the qualitative analyses were integrated in the ICF framework (World Health Organization, 2017) as a theoretical model in order to find a multifactorial construct about severity. Stage V was guided by the question ‘How can the SLTs’ views and the ICF codes be integrated in the SSDSC?’. An overview of Stage V is provided in Appendix IV.

#### Stage V analysis

Two final steps of the CGT methodology were completed:

- **Theoretical coding**: The themes and ICF severity codes were combined by theoretical coding and allocated to the ICF framework. The themes and ICF severity codes were included in the ICF domains by reading the specifications in the ICF manual (World Health Organization, 2017). When the content of a theme corresponded with the description of the ICF severity code, then the theme was added to that particular ICF domain.
- **Co‐construction of theory**: All outputs from the qualitative study were revisited. The themes and the ICF severity codes were summarised in one or two sentences. With the quotes from the focus group discussions and the definitions of the ICF severity codes in mind, an umbrella term was chosen. Themes allocated to Body Functions and Activities and Participation were named as severity factors. Personal and Environmental factors were labelled as facilitators and barriers.

An overview of Stage V is provided in Appendix IV.

## RESULTS

This five‐stage study resulted in a co‐created ICF‐based multifactorial SSDSC representing SLTs' views on severity factors, facilitators and barriers. The SSDSC can be used by SLTs in daily practice. An overview of the final result is provided in Appendix V. The main findings from all stages of this study are described below.

### Stage I results

The severity inventory yielded 139 severity indicators of SSD. Duplicates were merged, resulting in a list of 111 unique items, (e.g., the size of the phonemic repertoire, the number of articulation errors, the intelligibility, how well the child is understood by acquaintances and strangers (functional intelligibility), the effect of therapy, the child's frustration). The severity indicators list was coded by the first author into 23 codes. During the classification assignment, the five pairs of SLTs formulated on average 13.5 potential themes worded in 67 unique phrases (Table 4).

**TABLE 4:** SLT characteristics, number of potential themes, and frame of reference during the classification assignment (*n* = 10).

ID	Pseudonym	Potential themes	Frame of reference
Pair #1	Denise	15	Impact on the child and impact on the social environment
	Karin		
Pair #2	Anna	17	Inspired by PROMPT‐principles for motor learning
	Sandra		
Pair #3	Lisa	8	SLT's assessment and impact on daily functioning
	Jolanda		
Pair #4	Iris	18	ICF framework
	Carolien		
Pair #5	Merel	10	SLT's assessment and impact on daily functioning
	Esther		

Abbreviations: ICF, International Classification of Functioning, Disability and Health; SLT, speech‐language therapists.

The 67 unique phrases were reduced to eight potential themes by interpretation of their meaning. Consensus between pairs of SLTs was checked by comparing potential themes with the underlying severity indicator terms. In many cases the SLT pairs had allocated the same terms to the same potential themes. For example ‘*persistence in response to therapy* [hardnekkigheid nav therapie]’ or ‘*persistence* [hardnekkigheid]’ represented the same severity indicators. In contrast, some potential themes such as ‘*expectations* [verwachtingen]’ and ‘*environmental factor* [omgevingsfactor]’ were discussed in the consensus workshop, because the pairs did not reach consensus. A total of eight potential themes covered 71 severity indicators; 40 additional severity indicators were not allocated so were discussed in the consensus workshop (Table 5).

**TABLE 5:** Stage I‐III outcomes qualitative analyses: Themes and codes.

Stage	Activity	Participants	Themes/codes	Indicators included	Indicators not included
I, TA3	Classification assignment	68	Potential Themes:	71	40
			1. Intelligibility		
			2. Communicative participation		
			3. Emotional burden or hinderance		
			4. Speech characteristics		
			5. Persistence		
			6. Environmental factors		
			7. Impact on general development		
			8. Behaviour		
I, TA4	Consensus workshop	13	Reviewed Themes:	104 (+ 33)	7
			1. Intelligibility		
			2. Communicative participation		
			3. Emotional burden or hinderance		
			4. Speech characteristics		
			5. Persistence		
			6. Environmental factors		
			7. Impact on general development		
II, CGT3	Focus Group	10	Focused Codes:	104	7
			1. Intelligibility		
			2. Communicative participation		
			3. Child's perspective		
			4. Speech accuracy		
			5. Persistence		
			6. Environmental factors		
			7. Impact		
			8. Concomitant factors		
			9. Professional point of view		
III, TA5	TA		Themes:	104	7
			1. Intelligibility		
			2. Communicative participation		
			3. Child's perception		
			4. Speech accuracy		
			5. Persistence		
			6. Environmental factors		
			7. Impact		
			8. Concomitant factors		
			9. Professional point of view		

Abbreviations: CGT, constructivist grounded theory; TA, thematic analysis.

During the consensus workshop 33 out of the 40 remaining severity indicators were assigned to a potential theme based on 100% consensus. Consensus was not reached on seven severity indicators; the potential themes thus covered 104/111 severity indicators. Finally, the result of the TA of the severity indicators list was summarised in seven reviewed themes: (1) *intelligibility* [verstaanbaarheid], (2) *communicative participation* [communicatieve redzaamheid], (3) *emotional burden or hinderance* [emotionele last], (4) *speech characteristics* [spraakkenmerken], (5) *persistence* [hardnekkigheid], (6) *environmental factors* [omgevingsfactoren], and (7) *impact on general development* [invloed overige ontwikkeling]. No severity indicators were allocated to potential theme (8) *behaviour* [gedrag], and was therefore removed from the list (Table 5).

### Stage II results

The CGT analysis of the data from the focus group in Stage II resulted in a set of nine focused codes: (1) *intelligibility*, (2) *communicative participation*, (3) *child's perspective*, (4) *speech accuracy*, (5) *persistence*, (6) *environmental factors*, (7) *impact*, (8) *concomitant factors*, and (9) *professional point of view* (Table 5). Each focused code will be explained and illustrated consecutively.

#### Intelligibility

Intelligibility was discussed frequently as an indicator of severity. Anna said she used the ICS in her daily practice for evaluation of the severity, and others described context (e.g., home, school) as an important influence on intelligibility and severity. Merel agreed: ‘Intelligibility does fit into the picture whether a speech disorder is really severe or not’.

#### Communicative participation

Participation as a severity indicator was widely shared amongst the participants. Anna: ‘Severity depends on participation, for sure’ (all the other participants nodded in agreement). In the reflective discussion, there was reflection on the difference between the concepts of ‘intelligibility [verstaanbaarheid]’ and ‘communicative participation [communicatieve redzaamheid]’. Those words were often combined in the participants' answers, but the differences between the two remained unclear. Sandra explained the way she indirectly evaluated participation during assessments using the ICS saying ‘it shows how much the child relies on his mother as an interpreter’. Therefore, the scope of both concepts was discussed and it was suggested that unintelligible speech may be a barrier to communicative participation. However, Lisa, Esther, and Sandra recommended that intelligibility and communicative participation should be treated as separate entities.

#### Child's perspective

Dutch speech therapists have a common term for the child's perspective ‘de last die kind ervan heeft’ that literally translates as ‘the burden for the child’. *Burden* has an emotional implication regarding how children perceive their problems, as well as a practical implication when they determine how much having SSD restricts everyday experiences. For example, Karin stated: ‘Well, what is most important with respect to severity, actually, is the burden [de last] for the child’. The child's own burden was identified as playing a prominent role in severity, so the child's perspective was identified as an important severity indicator.

#### Speech accuracy

The SLTs had complete agreement that speech accuracy was an important indicator of severity of SSD. Merel, for example, evaluated children's phoneme inventory and determined ‘which sounds are acquired, which sounds are being substituted, whether there are typical processes or atypical processes’. The value of comparing the child's speech to TD was important. All participants agreed with Jolanda who said that ‘the severity of the disorder on its own, should be compared to typical phonological development’. Severity also depended on the complexity of the speech problem. Highly restricted phoneme repertoires or atypical phonological processes were mentioned as severity indicators.

The complexity of SSD as a severity indicator was also recognised and elaborated on during the reflective discussion. Carolien for example described complexity as ‘many different types of errors’, as well as the complexity of the features of a specific sound. Additionally, ‘speech motor problems’ were mentioned as an essential indicator of severity by Karin, Sandra, and Carolien. After extensive discussion about speech accuracy, all SLTs agreed that a detailed description of the child's speech development and the nature of the speech errors were important to determine severity of SSD.

#### Persistence

Persistence [hardnekkigheid] was identified as one of the most important indicators for a severity measure, but there was discussion about the scope of the concept. Sandra and Anna had different interpretations of persistence in the sense of progress during therapy on the one hand, and the stimulability of the sounds after instruction on the other hand. Sandra linked persistence to the stimulability of a single sound in response to teaching instructions. Anna, however, linked persistence to therapy progress over time. Carolien explained how hard it is to determine the severity at the start because it also depended on the progress over time: ‘Sometimes, at the start, when I hear the child, I think ‘wow!’ (impressed). But, after 3 weeks of therapy, I think ‘huh?’ (surprised)]. In that case the severity is not so bad!’. ‘Persistence’, understood as the progress over time during therapy, was mentioned by Anna, Carolien, and Karin as an essential indicator in a severity measure.

#### Environmental factors

Participants mentioned that barriers experienced by families, friends and the children were important indicators of severity. Merel suggested that participation depends on ‘what is required’ and on ‘environmental support’. Lisa agreed, but Iris tried to stimulate the discussion by asking if communicative competence isn't ‘just a sum of all those things together?’ which was ultimately endorsed by all. The discussion illustrated that important severity factors included the role and support from the community and the children's perspective.

#### Impact

The impact of SSD was also recognised as an important indicator in the reflective discussion. Impact is a broad concept for which different words were used. Anna explained that she distinguished between ‘the impact [gevolgen] on the child's social development’ and ‘just [gewoon] general communicative participation’. Impact also included the impact of SSD on the child's personality, as Sandra explained: “Yes, the consequences for the child themself! Interpersonal factors. In the intake, I always ask the mother to describe your child in five factors, ‘how Pietje is’, that is about personality and I think this is the same category”.

#### Concomitant factors

The role of concomitant factors unrelated to speech, such as behaviour, intelligence, and other external factors, was identified as missing from the severity indicators list. Discussing the missing items, Karin stated that ‘severity will be determined by several indicators, but they are not in there. Look, when a child is less intelligent…’. Other concomitant indicators related to speech included ‘auditory attention’; ‘telescoping’ or ‘speech rate’ were mentioned as missing on the severity indicators list.

#### Professional point of view

The determination of severity was described as coming from a subjective interpretation of speech characteristics based on clinical experience. Jolanda, for example, mentioned her ‘clinical ears’ to determine severity. Subjectivity was recognised and confirmed by Sandra saying: ‘no matter how you slice it, what I do, is subjective’. She explained that the professional point of view is mainly based on ‘your own experience, thinking that this child has a serious problem or that child I will be done with soon. Let's say practice‐based, let's call it that’. The other participants nodded. The need for normed assessment instruments was recognised by all participants; however, these are scarce in The Netherlands.

### Stage III results

In Stage III the reviewed themes from Stage I and the focused codes from Stage II were merged and summarised in nine Themes (Table 5). (1) *Intelligibility* and (2) *communicative participation* were included unchanged in the final list of themes. Reviewed theme *emotional burden or hinderance* and focused code *child's perspective* both reflected how children perceive their own speech so the theme was named as (3) *child's perception*. Reviewed theme *speech characteristics* and focused code *speech accuracy* also represented the same content and were merged into theme (4) *speech accuracy*. (5) *Persistence*, and (6) *environmental factors* were retained unchanged and added to the list of themes. Reviewed theme *impact on general development*, and focused code *impact* also reflected similar content and were merged into the theme (7) *impact*. Finally, focused codes (8) *concomitant factors*, and (9) *professional point of view* had no equivalent on the list of reviewed themes, so were added.

### Stage IV results

In Stage IV the items from the severity indicators list were compared to the selected speech‐related ICF codes from the ICF browser (World Health Organization, 2017). The deductive analysis resulted in a selection of ICF severity codes, retrieved from three domains in the ICF model (Table 6). First, Body Function codes were chosen from b1 Mental functions, and b3 Voice and speech functions. From the domain Activities and Participation, codes were included from d1 Learning and applying knowledge, d3 Communication, d7 Interpersonal interactions and relationships, d8 Major life areas, and d9 Community, social and civic life. Finally, Environmental Factor codes were selected from e3 Support and relationships, and e4 Attitudes. There were no codes from the ICF domain Body Structures (s‐codes) matching the severity indicators. Personal Factors are not coded in the ICF.

**TABLE 6:** Stage IV outcomes CA selection: Selected ICF codes.

Stage	Activity	Themes/codes
IV, CA2	CA	b1 Mental functions
	ICF Body Functions	b117 Intellectual functions
		b122 Global psychosocial functions
		b126 Temperament and personality functions
		b1266 Confidence Mental functions
		b152 Emotional functions
		b1522 Range of emotion
		b156 Perceptual functions
		b164 Higher‐level cognitive functions
		b1643 Cognitive flexibility
		b3 Voice and speech functions
		b320 Articulation functions
	CA	d1 Learning and applying knowledge
	ICF Activities and Participation	d133 Acquiring language
		d3 Communication
		d330 Speaking
		d7 Interpersonal interactions and relationships
		d710 Basic interpersonal interactions
		d720 Complex interpersonal interactions
		d8 Major life areas
		d820 School education
		d9 Community, social and civic life
		d940 Human rights
		d999 Community, social and civic life
	CA	e3 Support and relationships
	ICF Environmental Factors and Personal Factors	e310 Immediate family
		e320 Friends
		e325 Acquaintances, peers, colleagues, neighbours, and community members
		e340 Personal care providers and personal assistants
		e345 Strangers
		e398 Support and relationships
		e4 Attitudes
		e410 Individual attitudes of immediate family members
		e450 Individual attitudes of health professionals
		e455 Individual attitudes of other Professionals
		e498 Attitudes

Abbreviations: CA, content analysis; ICF, International Classification of Functioning, Disability and Health.

### Stage V results

The nine themes From Stage III and the ICF severity codes were placed in the ICF model resulting in the SSDSC representing SLTs’ views on the severity of SSD (Table 7). The study yielded nine severity themes: (1) *intelligibility*, (2) *speech accuracy*, (3) *persistence*, (4) *the child's perception*, (5) *impact*, (6) *communicative participation*, (7) *concomitant factors*, (8) *professional point of view*, and (9) *environmental factors* (Table 7). Theme (1) *intelligibility* comprised both intelligibility in the sense of accuracy (sounds recognised in words), and intelligibility in context (message recognised and understood in different situations).

**TABLE 7:** Stage V outcomes CGT: Themes and ICF domains.

Stage	Themes/codes
V, CGT4	Themes body functions:
	1. Intelligibility
	2. Speech accuracy
	3. Persistence
	4. The child's perception
	5. Impact
	7. Concomitant factors
	Themes activities and participation:
	1. Intelligibility
	5. Impact
	6. Communicative participation
	Themes environmental and personal factors:
	1. Intelligibility
	5. Impact
	8. Professional point of view
	9. Environmental factors

Abbreviations: CGT, constructivist grounded theory; ICF, International Classification of Functioning, Disability and Health.

SLTs indicated that not all themes were of equal value. Therefore, the results are presented in terms of *Main severity factors*, *Other severity factors* and *Facilitators and barriers for severity*. The *Main severity factors* lean on strong consensus during focus group discussions, representing the child's functioning in the domains of Body Functions and Participation: (I) *Speech accuracy*, (II) *The child's perception of the impact of their speech*, and (III) *Intelligibility in communication*.

The *Other severity factors* were mentioned during the focus group discussions but less importance was attached to them. These Other severity factors were (1) *Concomitant factors* in the domain of Body Functions, and (2) *Impact* in the domain of Participation.

Additionally, the themes from the domain Environmental and Personal factors were summarised in two *Facilitators and barriers for severity*: (a) *Support* and (b) *Expertise*. The results from Stage V were summarised in Table 8, and discussed below.

**TABLE 8:** Stage V outcomes CGT: Main severity factors, other severity factors, and facilitators and barriers for severity.

Stage	Themes/codes
V, CGT5	Main severity factors:
	I. *Speech accuracy*
	II. *Child's perception of the impact of their speech*
	III. *Intelligibility in communication*
	Other severity factors
	1. *Concomitant factors*
	2. Impact
	Facilitators and barriers for severity:
	a. *Expertise*
	b. *Support*

Abbreviation: CGT, constructivist grounded theory.

**FIGURE 1:**
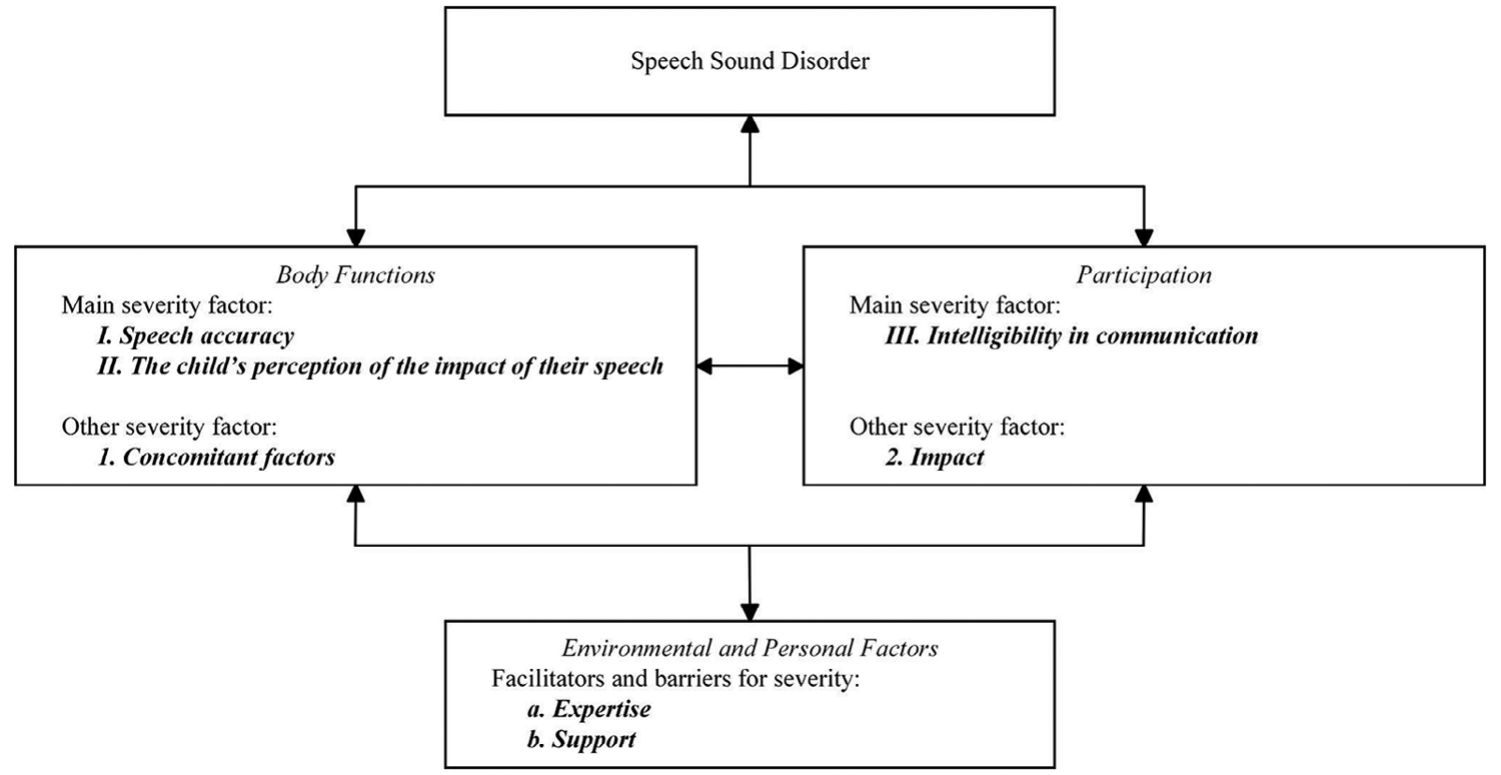
SSDSC: Severity factors in the ICF framework. ICF, International Classification of Functioning, Disability and Health; SSDSC, speech sound disorder severity construct.

All factors, facilitators and barriers and their place in the ICF model were depicted in the SSDSC (Figure 1).

#### Main severity factors

- *Speech accuracy*: Speech accuracy was related to the ICF code of Articulation functions (b320) and it included the themes (1) *intelligibility*, (2) *speech accuracy*, and (3) *persistence*. It defines how speech development can be described in terms of accuracy, for example PCC, phonological patterns, or percentage of words understood, and also the progress over time. These terms arose frequently during all phases of the study and were confirmed by SLTs as contributing to the severity of SSD.
- *The child's perception of the impact of their speech*: The child's perception of their speech was related to the ICF codes for Mental functions (b1), and Emotional functions (b152) which are about development and the consequences for learning, social and emotional development. This severity factor comprised the themes (4) *the child's perception* and (5) *impact*. These themes are about how children cope with their speech problems, and which emotions are involved. It also comprised the emotional burden ['de last die het kind ervan heeft']. *The child's perception of the impact of their speech* was highlighted as a theme in all stages of the study.
- *Intelligibility in communication*: The themes (1) *intelligibility* in sense of intelligibility in context and (6) *communicative participation* are closely related in children with SSD and were both included in the third main severity factor. It comprises communication with people in close relationships as well as friends and strangers. In the ICF browser it is described as d710 Basic interpersonal interactions and d720 Complex interpersonal interactions. There was strong consensus amongst the SLTs in different stages of the study about these themes in relation to the severity of SSD, but there is no clear definition of the difference between both themes.

#### Other severity factors

- *Concomitant factors*: This first other severity factor comprises theme (7) *concomitant factors* as a Body Function representing ICF codes from b1 Mental functions and b3 Voice and speech functions. It is related to cognitive functioning, learning and other developmental areas besides speech and communication that may impede the child's development. Concomitant factors were discussed in the focus group when evaluating the SLTs’ experience in diagnosing SSD. It is important for a holistic view of the child's functioning, but is not on ‘top of mind’ when considering severity.
- *Impact*: This second other severity factor includes theme (5) *impact* at the domain of Participation. It is about learning and applying knowledge in major life areas such as school education and functioning at home or with friends, representing ICF codes d1 Learning and applying knowledge and d820 School education. *Impact* also relates to human rights in the sense of having equal access to all aspects of daily life compared to peers, in ICF codes d9 Community, social and civic life.

#### Facilitators and barriers for severity: Environmental and personal factors

Environmental and Personal Factors serve as facilitators or barriers for the functioning of the child and illustrate the context the child is living in. A facilitator can relieve the participation problems of the child, while a barrier can worsen the participation problems or even the disorder in the domain of Body Functions.

- *Expertise*: Theme (8) *professional point of view* was included in facilitators and barriers for severity *Expertise*, related to ICF codes e4 Attitudes. It is related to SLTs’ skills and knowledge about SSD. It may be a facilitator when the SLT is well experienced in diagnosing and treating SSD. But, it may be a barrier when misdiagnosis results in inadequate therapy which may hinder the child from achieving acceptable communication, socialisation, and learning.
- *Support*: The Themes (1) *intelligibility*, (5) *impact* and (9) *environmental factors* were comparable in content and were included in the facilitators and barriers for severity *Support*. It involves personal relationships and community understanding as described in ICF codes e3 Support and relationships. Familiarity with the child's speech will support the child and may facilitate the child's communication. It is also related to the attitudes from teachers and other professionals and their willingness to support the child. *Support* may act as a barrier when the community is not supportive, or, on the other hand, as a facilitator when the child experiences support.

## DISCUSSION

In order to develop a multi‐factorial severity measure for SSD in the future, the purpose of this five‐step study was to examine how SLTs determine the severity of SSD in children, and how their perspectives were consistent with the ICF. A total of 91 of Dutch SLTs were asked about their views and experiences in daily practice. When evaluating severity of SSD, three main severity factors were identified: (I) *Speech accuracy*, (II) The *child's perception of the impact of their speech*, and (III) *Intelligibility in communication*. Other Severity factors were (1) *Concomitant factors*, and (2) *Impact*. Additionally, facilitators or barriers for severity were (a) *Expertise*, and (b) *Support* from the family, teachers and community. The severity factors, and facilitators and barriers are summarised in the SSDSC. The SSDSC may provide a framework for the development of a severity measure for daily practice, which will need to be further tested and validated in the future.

The results revealed two main points. First, even though SLTs have tacit and explicit knowledge about the severity of SSD in children, the results from the study showed that severity is not easy to define. The participating SLTs did not have a uniform way to determine the severity of SSD. The SLTs’ explicit knowledge about severity was more clearly articulated regarding the Theme of *speech accuracy*. For example, SLTs described severity in comparison with TD and during the assessment of motor speech problems. The focus group discussions revealed tacit knowledge stimulating the SLTs to consider their common practices and share their experiences determining severity of SSD. *Concomitant factors* and the *professional point of view* were new themes which emerged from the discussions.

Second, and in line with Flipsen et al. (2005), the current study showed that severity of SSD is a multifactorial concept. The focus group discussion revealed that multiple factors are taken into account when SLTs determine severity of SSD and there was strong consensus between the participants on the factors that should be evaluated. The severity factors and the facilitators and barriers identified in the present study mapped onto the domains of the ICF framework.

Compared to the use of a holistic TOM, as suggested by Enderby et al. (2013), the transparency of the severity factors makes it possible to start thinking about specific instruments to measure severity, thus objectifying it. The use of standardised and validated instruments makes the assessment of severity less dependent on the user, and therefore less subjective than a TOM. In addition, the use of a TOM is based on professional judgement, whereas the inclusion of multiple views, such as the judgement of parents and children themselves, as well as the judgement of SLTs, is important. As Zerbeto et al. (2023) showed that children had different perceptions on ICF domains before and after therapy, it is important to include children's perceptions of their own speech when determining the severity of SSD.

### Speech accuracy

During the focus group discussions, *intelligibility* was recommended as a main severity factor. Intelligibility was linked to both Body Functions and Activities and Participation (McLeod & Threats, 2008), showing that intelligibility depends on the speaker as well as the listener. These findings are in line with Miller (2013) who recommended using ‘signal‐dependent’ and ‘signal‐independent’ intelligibility measures in diagnosing SSD. Signal‐dependent intelligibility is associated with speech characteristics, and is covered by main severity factor speech accuracy.

There was strong consensus between participants about *speech accuracy* being a main severity factor at the Body Functions domain. Many researchers have demonstrated that PCC is a reliable outcome measure for speech accuracy (Garrett & Moran, 1992; Newbold et al., 2013; Shriberg & Kwiatkowski, 1982). However, the SLTs in the current study did not have a uniform way of determining accuracy. In daily practice, their judgement about accuracy was based on the deviation from adult speech and a comparison with TD, rather than calculation of PCC.

*Persistence* was also rated as an important severity factor; however, it is not possible to evaluate persistence at the beginning of intervention. Therefore, in the severity model, *persistence* is not designated as a separate main severity factor.

### The child's perception of the impact of their speech

The second main severity factor that was mentioned frequently was *the child's perception of the impact of their speech*, including the social and emotional impact for the child. The burden for the child [de last die een kind ervan heeft] was mentioned by the participants when creating the severity indicators inventory as well as during the focus group discussions. The participants agreed that the child's perception of their SSD was an important severity factor covering both themes *the child's perception* and *impact*. *The child's perception of the impact of their speech* therefore, is related to personal characteristics, such as temperament and personality functions which may determine how the child copes with the SSD. However, the ICF category of mental functions also includes emotional functions that comprises feelings such as insecurity and fear (World Health Organization, 2017), that may develop as a result of the experience of failure. Consequently, as children get older and become more aware of their speech, they may withdraw from social situations and become isolated.

### Intelligibility in communication

*Intelligibility* is associated with characteristics of the listener, speaker and context and is closely related to communicative participation of children with SSD (Weismer, 2008). This fits the description of a ‘signal‐independent’ intelligibility measure in diagnosing SSD cited by Miller (2013). Signal‐independent intelligibility is associated with verbal information other than speech, and non‐verbal information. *Intelligibility (in context)* and *communicative participations* were terms mentioned frequently by almost all SLTs, and often reported as a first response. However, the discussions in the focus group and also during the consensus workshop, could not clarify whether there was a difference between *intelligibility in context* and *communicative participation*. Therefore, *Intelligibility in communication* is chosen as a main severity factor representing both in the ICF Participation domain.

### Concomitant factors

*Concomitant factors* were determined to influence severity of SSD, but not be a main factor. *Concomitant factors* such as general learning problems or DLD may influence the child's functioning. Recent literature supports the association between concomitant factors and the SSD severity (Iuzzini‐Seigel et al., 2022; Torres et al., 2020). For example, for the combination of SSD and DLD, it has been suggested that motor constraints limit the construction of speech sequences, which may affect expressive grammar. Conversely, improved language skills may improve speech sequencing skills (Chilosi et al., 2022).

### Impact

*Impact* is seen as the practical effect of the main severity factor *The child's perception of the impact of their speech*. In accordance with McCormack, McLeod, Harrison, et al. (2010) and Feeney et al. (2012), it is likely that *Intelligibility in communication* and *The child's perception of the impact of their speech* are closely related, and that understanding the impact of SSD on children, should assess numerous domains of functioning.

### Expertise and Support

*Expertise* and *Support* as *Facilitators and barriers for severity* are closely related to the individual attitudes and help of, for example, immediate and extended family, friends, community members, people in positions of authority (e.g., teachers) and health professionals, including SLTs (World Health Organization, 2017). The attitude and support from the environment, for example, how willing a teacher is to listen and to take time to understand a child with SSD (McLeod et al., 2013), may be a facilitator or barrier to communication success. Therefore, *Expertise* may be most related to the main severity factor *Speech accuracy*. Appropriate interventions for SSD help the child to expand their phoneme repertoire and develop intelligible speech. *Support* may therefore be most relevant to the main severity factor *Intelligibility in communication*. *Expertise* and *Support* can make a difference in the lives of children with SSD and can influence the severity of SSD.

## LIMITATIONS

Because SLTs with various backgrounds and expertise were included in this study, the conclusions can be applied to many children with SSD as defined by the International Expert Panel on Multilingual Children's Speech (IEPMCS, 2012, p. 1). However, it is important to note that this study reflects the views of SLTs in The Netherlands. The SSDSC, therefore, may be culture‐specific. In the Netherlands, SLTs are trained to work with the ICF model, which makes it a framework of thought that cannot be ignored. This may have guided their thinking during the interviews, discussions and activities. However, because the ICF is an WHO international framework, the SSDSC should be applicable in all cultures where working in line with the principles of the ICF model is common.

## CLINICAL IMPLICATIONS

The present findings show that SLTs consider multiple factors when determining the severity of SSD. Determining the severity of SSD aids SLTs in selecting the children who are in need of help. Adequate evaluation of speech accuracy, intelligibility and participation and the child's perception of its speech problem are all elements SLTs consider doing speech assessments.

The current study also showed that the severity factors are related to TD. SLTs frequently mentioned ‘deviation from age norms’, and ‘the comparison to typical speech development’. These findings support the idea of McLeod & Crowe (2018), to relate PCC to the age of the child because of the developmental nature of speech accuracy. The SLTs in the current study concluded that they are in need for reliable, standardised, validated, and normed instruments for all severity factors. This is in line with Fabiano‐Smith (2019) who recommended the use of standardised tests for more accurate diagnoses of SSD.

While more research is needed to determine how to evaluate severity factors, this study shows what factors SLTs might consider when determining the severity of SSD. On the basis of these findings, SLTs may wish to review their practice and consider how at least the main severity factors Speech accuracy, The child's perception of the impact of their speech, and Intelligibility in communication, can be incorporated into a speech assessment.

## CONCLUSION

In conclusion, the current study emphasises that the severity of SSD, as summarised in the SSDSC, is a multifactorial construct. SLTs consider three main severity factors when determining the severity of an SSD: (I) *Speech accuracy*, including *intelligibility, speech accuracy*, and *persistence*, (II) *The child's perception of the impact of their speech*, comprising *the child's perception* and *impact*, and (III) *Intelligibility in communication*, including *intelligibility* and *communicative participation*. Other severity factors were (1) *Concomitant factors* and (2) *Impact*, considered when they were very prominent. Finally, the contribution of (a) *Expertise* and (b) *Support* as facilitators or barriers for severity were taken into account when determining the severity of SSD. This study highlights the need for SLTs to rethink how they think about severity as a simplistic construct reflecting only speech accuracy. It is recommended that a broader holistic approach to measuring severity is adopted. Continued research to test the severity factors is needed, preferably in a quantitative design.

## CONFLICT OF INTEREST STATEMENT

The authors certify that they have no financial, or non‐financial interest in the subject matter or materials discussed in this manuscript. The third author is a co‐author of the Intelligibility in Context Scale, and the first and last author are translators of the Intelligibility in Context Scale‐Dutch.

## Supporting information



Supporting Information

Supporting Information

Supporting Information

Supporting Information

Supporting Information

## Data Availability

The data that support the findings of this study are available from the corresponding author upon reasonable request.
