# Supplementary material for: Speech and language therapists' insights into severity of speech sound disorders in children for developing the speech sound disorder severity construct
Source: Int J Lang Commun Disord. 2025 Mar 26;60(3):e70022. doi: 10.1111/1460-6984.70022 (PMC11946931; doi:10.1111/1460-6984.70022)
Supplement: Supplementary file 1 — Supporting Information [file JLCD-60-0-s003.pdf]

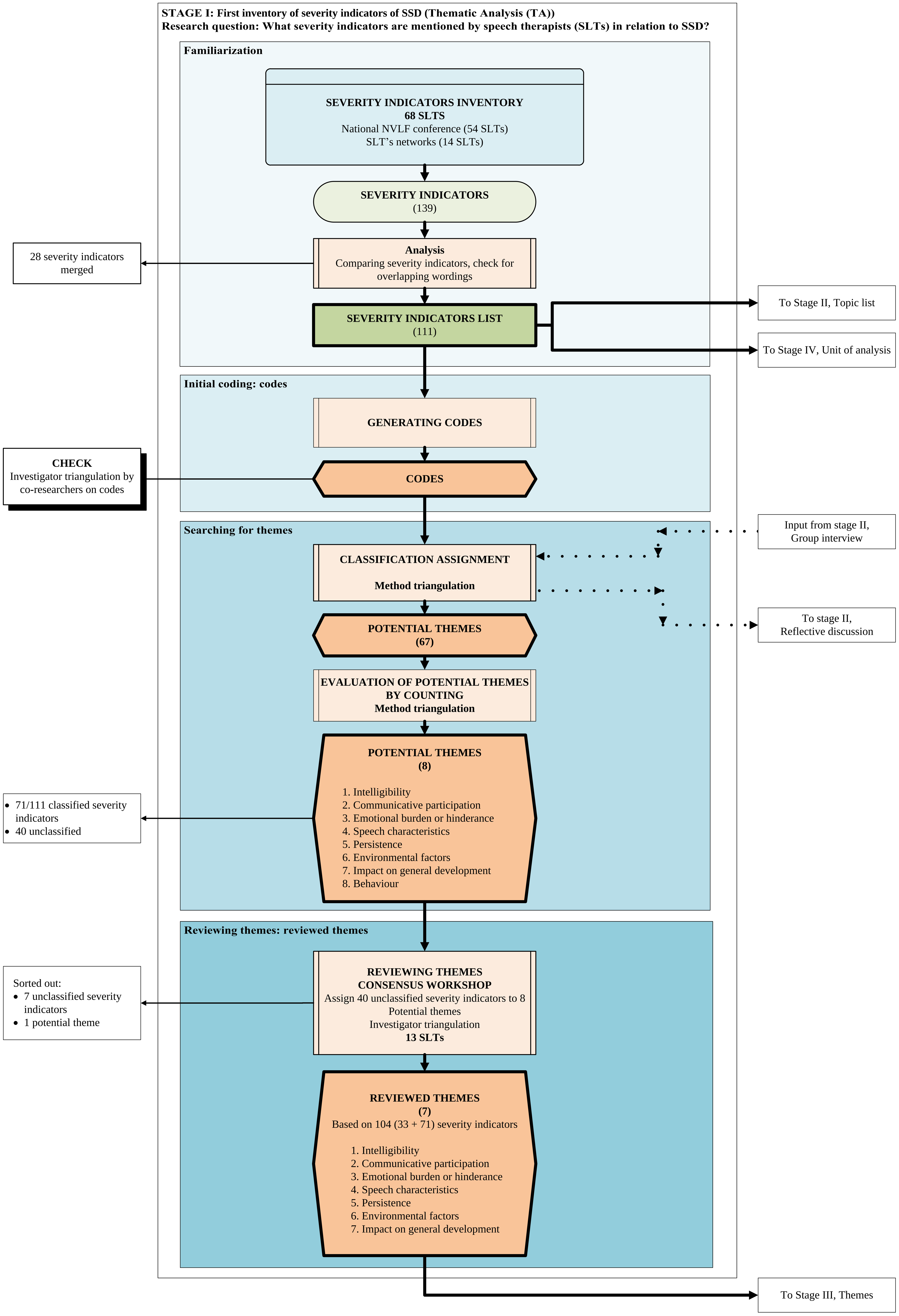
STAGE I: First inventory of severity indicators of SSD (Thematic Analysis (TA))
Research question: What severity indicators are mentioned by speech therapists (SLTs) in relation to SSD?
Familiarization
SEVERITY INDICATORS INVENTORY
68 SLTS
National NVLF conference (54 SLTs)
SLT's networks (14 SLTs)
SEVERITY INDICATORS
(139)
28 severity indicators merged
Analysis
Comparing severity indicators, check for overlapping wordings
SEVERITY INDICATORS LIST
(111)
To Stage II, Topic list
To Stage IV, Unit of analysis
Initial coding: codes
GENERATING CODES
CHECK
Investigator triangulation by co-researchers on codes
CODES
Searching for themes
Input from stage II, Group interview
CLASSIFICATION ASSIGNMENT
Method triangulation
To stage II, Reflective discussion
POTENTIAL THEMES
(67)
EVALUATION OF POTENTIAL THEMES BY COUNTING
Method triangulation
POTENTIAL THEMES
(8)
1. Intelligibility
2. Communicative participation
3. Emotional burden or hinderance
4. Speech characteristics
5. Persistence
6. Environmental factors
7. Impact on general development
8. Behaviour
• 71/111 classified severity indicators
• 40 unclassified
Reviewing themes: reviewed themes
Sorted out:
• 7 unclassified severity indicators
• 1 potential theme
REVIEWING THEMES
CONSENSUS WORKSHOP
Assign 40 unclassified severity indicators to 8 Potential themes
Investigator triangulation
13 SLTs
REVIEWED THEMES
(7)
Based on 104 (33 + 71) severity indicators
1. Intelligibility
2. Communicative participation
3. Emotional burden or hinderance
4. Speech characteristics
5. Persistence
6. Environmental factors
7. Impact on general development
To Stage III, Themes
