# Supplementary figures and images for: Speech and language therapists' insights into severity of speech sound disorders in children for developing the speech sound disorder severity construct
Source: Int J Lang Commun Disord. 2025 Mar 26;60(3):e70022. doi: 10.1111/1460-6984.70022 (PMC11946931; doi:10.1111/1460-6984.70022)

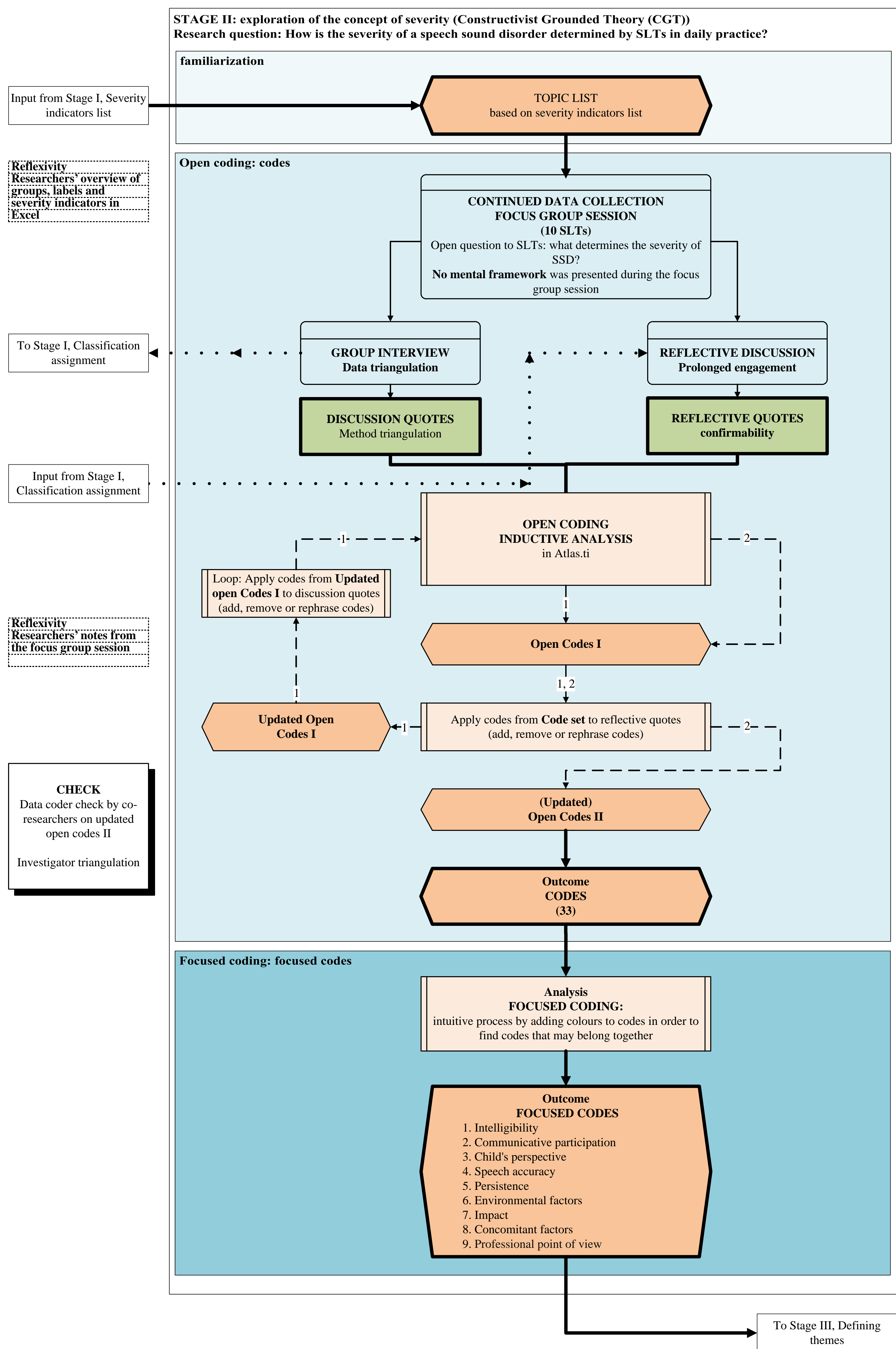

Supplement: Supplementary file 2 — Supporting Information [file JLCD-60-0-s005.pdf]

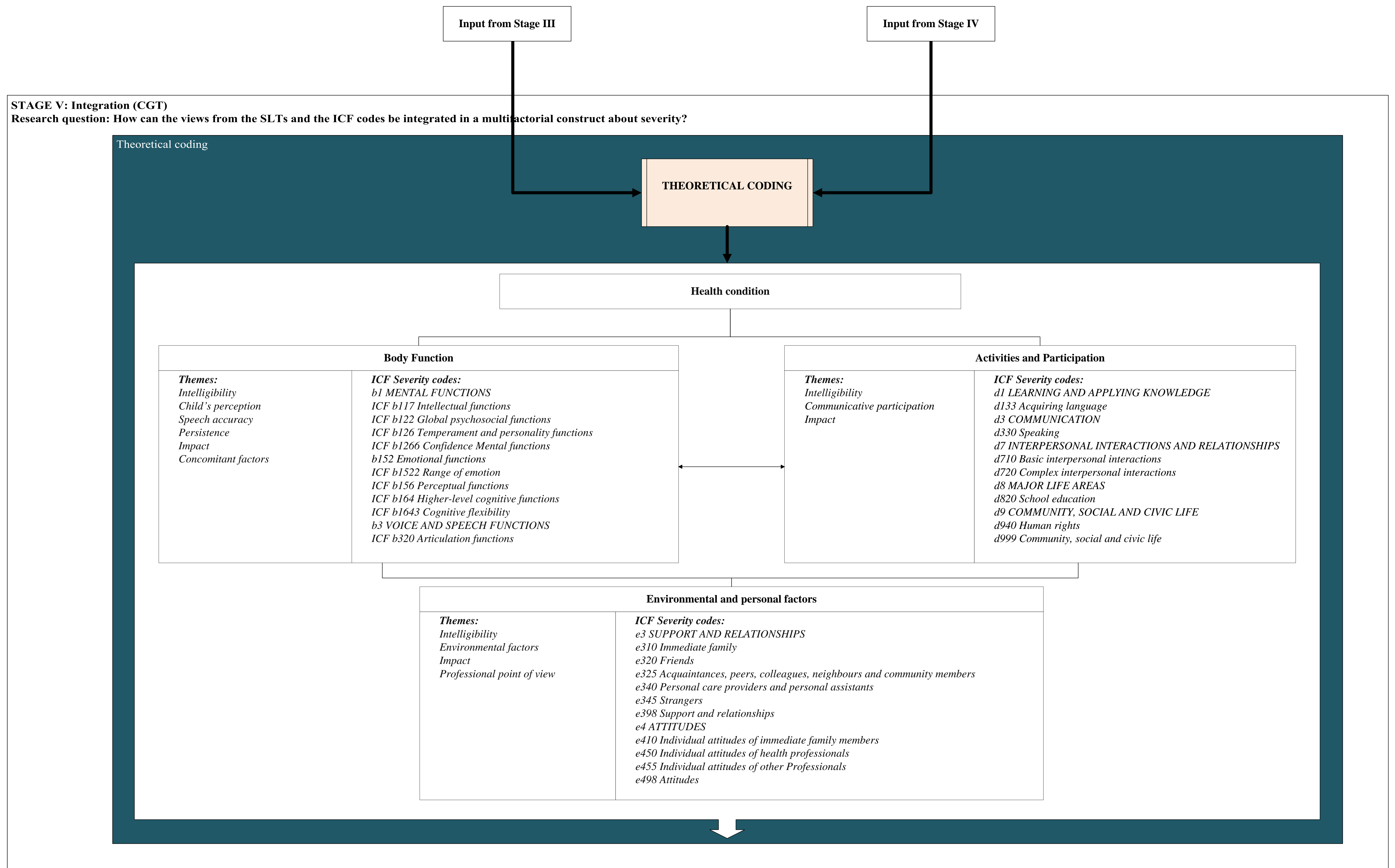

Supplement: Supplementary file 4 — Supporting Information [file JLCD-60-0-s002.pdf]
