# Supplementary material for: Speech and language therapists' insights into severity of speech sound disorders in children for developing the speech sound disorder severity construct
Source: Int J Lang Commun Disord. 2025 Mar 26;60(3):e70022. doi: 10.1111/1460-6984.70022 (PMC11946931; doi:10.1111/1460-6984.70022)
Supplement: Supplementary file 3 — Supporting Information [file JLCD-60-0-s004.pdf]

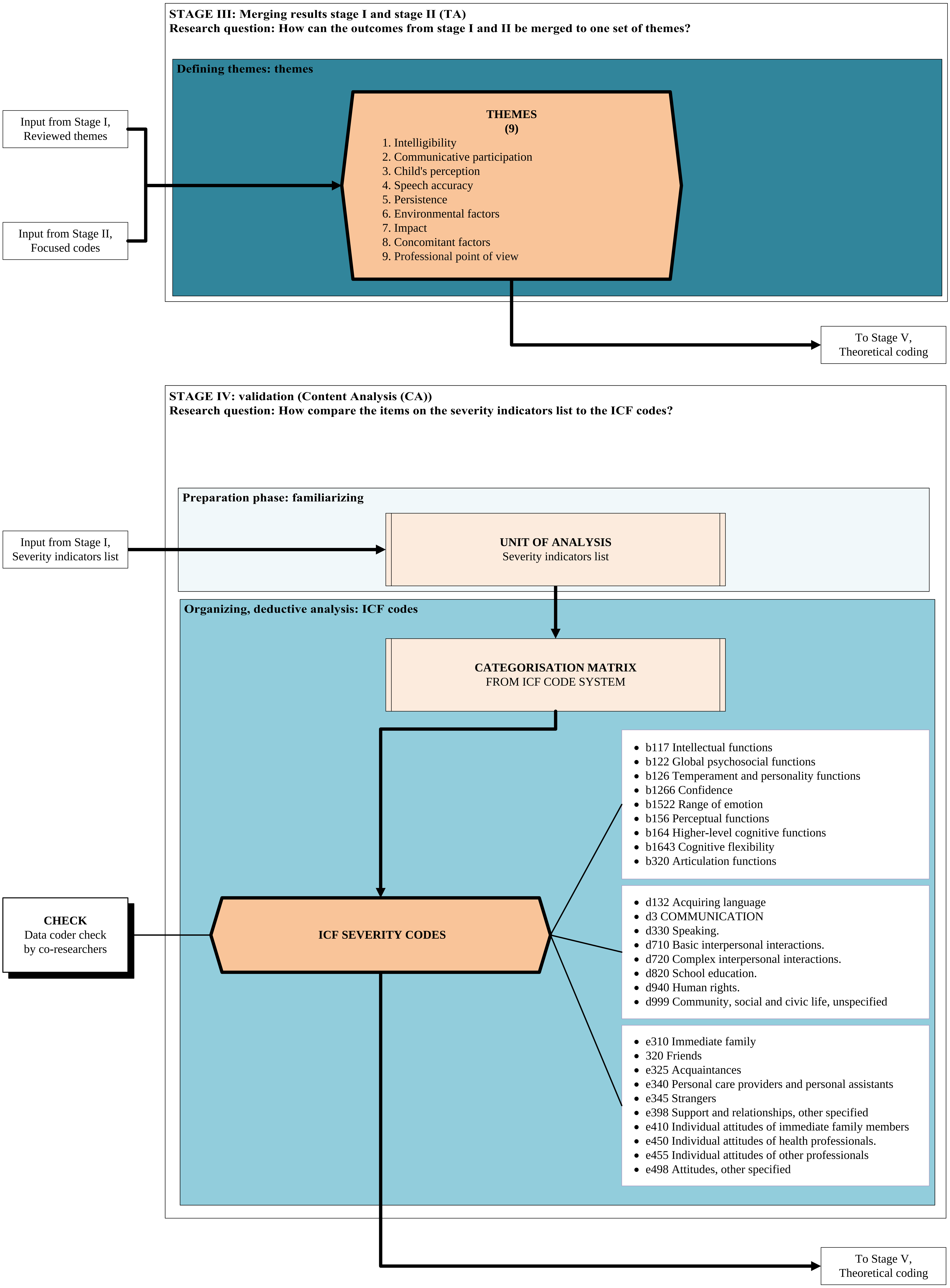
STAGE III: Merging results stage I and stage II (TA)
Research question: How can the outcomes from stage I and II be merged to one set of themes?
Defining themes: themes
Input from Stage I, Reviewed themes
Input from Stage II, Focused codes
THEMES
(9)
1. Intelligibility
2. Communicative participation
3. Child's perception
4. Speech accuracy
5. Persistence
6. Environmental factors
7. Impact
8. Concomitant factors
9. Professional point of view
To Stage V, Theoretical coding
STAGE IV: validation (Content Analysis (CA))
Research question: How compare the items on the severity indicators list to the ICF codes?
Preparation phase: familiarizing
Input from Stage I, Severity indicators list
UNIT OF ANALYSIS
Severity indicators list
Organizing, deductive analysis: ICF codes
CATEGORISATION MATRIX
FROM ICF CODE SYSTEM
• b117 Intellectual functions
• b122 Global psychosocial functions
• b126 Temperament and personality functions
• b1266 Confidence
• b1522 Range of emotion
• b156 Perceptual functions
• b164 Higher-level cognitive functions
• b1643 Cognitive flexibility
• b320 Articulation functions
• d132 Acquiring language
• d3 COMMUNICATION
• d330 Speaking.
• d710 Basic interpersonal interactions.
• d720 Complex interpersonal interactions.
• d820 School education.
• d940 Human rights.
• d999 Community, social and civic life, unspecified
• e310 Immediate family
• 320 Friends
• e325 Acquaintances
• e340 Personal care providers and personal assistants
• e345 Strangers
• e398 Support and relationships, other specified
• e410 Individual attitudes of immediate family members
• e450 Individual attitudes of health professionals.
• e455 Individual attitudes of other professionals
• e498 Attitudes, other specified
CHECK
Data coder check
by co-researchers
ICF SEVERITY CODES
To Stage V, Theoretical coding
