# Supplementary material for: Speech and language therapists' insights into severity of speech sound disorders in children for developing the speech sound disorder severity construct
Source: Int J Lang Commun Disord. 2025 Mar 26;60(3):e70022. doi: 10.1111/1460-6984.70022 (PMC11946931; doi:10.1111/1460-6984.70022)
Supplement: Supplementary file 5 — Supporting Information [file JLCD-60-0-s001.pdf]

Input from stage V, Integration

Co-construction of theory: Speech Sound Disorder Severity Construct (SSDSC)

Speech sound disorder

Body Function

Main Severity factors:
I. Speech accuracy (Intelligibility, Speech accuracy, *and* ***Persistence)*** *defines how speech development can be described in terms of accuracy, e.g. PCC, phonological patterns, or percentage of words understood, and also the progress over time.*
II. The Child's perception of the Impact of their Speech *(****Child's perception*** *and* ***Impact)*** *relates to development and the consequences for learning, social and emotional development. It is about how children cope with their speech problems, and which emotions are involved.*

Other Severity factors:
1. Concomitant factors (Concomitant factors) *is related to cognitive functioning, learning and other developmental areas besides speech and communication that may impede the child's development.*

Activities and Participation

Main Severity factors:
III. Intelligibility in communication (Intelligibility in contexts, *and* **Communicative participation)** *comprises communication with people in close relationships as well as friends and strangers. It is described as basic and complex interpersonal interactions and relationships.*

Other Severity factors:
2. Impact *(****Impact****) is about learning and applying knowledge in major life areas such as school education and functioning at home or with friends. Impact also relates to human rights in the sense of having equal access to all aspects of daily life compared to peers.*

Environmental and personal factors

Facilitators and Barriers for Severity:
a. Expertise (Professional point of view) *is related to SLTs' skills and knowledge about SSD. It may be a facilitator when the SLT is well experienced in diagnosing and treating SSD. But, it may be a barrier when misdiagnosis results in inadequate therapy which may hinder the child from achieving acceptable communication, socialisation, and learning.*
b. Support (Intelligibility, Impact, and Environmental factors) *involves personal relationships and community understanding. Familiarity with the child's speech will support the child and may facilitate the child's communication. It is also related to the attitudes from teachers and other professionals and their willingness to support the child. Support may act as a barrier when the community is not supportive, or, on the other hand, as a facilitator when the child experiences support.*
